# Cognitive reserve, microstructural brain lesions, and cognitive function in dementia‐free adults in UK Biobank

**DOI:** 10.1002/alz.088055

**Published:** 2025-01-09

**Authors:** Xiaolei Han, Xin Huang, Yifeng Du, Chengxuan Qiu, yongxiang wang

**Affiliations:** ^1^ Shandong Provincial Hospital affiliated to Shandong First Medical University, Jinan China; ^2^ Shandong First Medical University, Jinan, Shandong China; ^3^ Aging Research Center, Karolinska Institutet and Stockholm University, Stockholm Sweden

## Abstract

**Background:**

Microstructural brain lesions have been linked to cognitive health. However, their combined effect and the role of the cognitive reserve (CR) remain unclear. We investigated the interplay of microstructural brain lesions and cognitive reserve in cognitive function in middle‐aged and elderly adults.

**Method:**

This study used data from a prospective cohort of 26,490 UK Biobank participants in the MRI substudy. Markers of microstructural brain lesions included fractional anisotropy (FA), mean diffusivity (MD), intracellular volume fraction (ICVF), orientation dispersion (OD) indices, and isotropic volume fraction (ISOVF), which were estimated using diffusion magnetic resonance imaging (dMRI). Processing speed, reasoning, attention, executive function, and memory were assessed with neuropsychological tests, from which composite scores were derived. The composite CR indicator was generated from early‐life education, midlife occupation, physical activity, and social activity and was dichotomized into low and high CR based on mean CR score. We used multivariable linear regression models to examine the relation between markers of microstructural brain lesions and cognitive function while taking into account the potential interaction (interplay) of brain lesions and CR on cognitive function.

**Result:**

Controlling for sociodemographic, vascular, and genetic factors, decreased FA and ICVF and increased MD, as markers of impaired integrity of white matter microstructure, were significantly associated with lower composite z‐score of processing speed, attention, and executive function. Reduced ISOVF was associated with higher z‐score of processing speed [multivariable‐adjusted β‐coefficient=‐0.03. 95% CI ( ‐0.041, ‐0.019), p<0.001]. In the stratified analysis by levels of CR, decreased FA and ICVF were significantly associated with lower z‐scores of reasoning and memory, respectively, only in participants with low CR.

**Conclusion:**

This study suggests that impaired integrity of white matter microstructure is associated with reduced processing speed, attention, and executive function and that high CR could modify the association of microstructural brain lesions with cognitive function (e.g., memory and reasoning).

